# 1040. Comparative Surge Data on SARS CoV-2 Variants in a Rural Community – A Virtual Hospital Experience

**DOI:** 10.1093/ofid/ofac492.881

**Published:** 2022-12-15

**Authors:** William A Costello, Anthony Frank, Regina Rhodes, Gandhari Loomis, Rahul Sampath

**Affiliations:** UNC Health Blue Ridge - Morganton, Morganton, North Carolina; UNC Health Blue Ridge, Morganton, North Carolina; UNC Health Blue Ridge, Morganton, North Carolina; UNC Health Blue Ridge, Morganton, North Carolina; UNC Health Blue Ridge, Morganton, North Carolina

## Abstract

**Background:**

UNC Health Blue Ridge is a nonprofit community teaching hospital with 145 staffed beds. The COVID-19 pandemic has challenged rural hospitals like ours with high occupancy and periodic surges. UNC Health Blue Ridge COVID Virtual Hospital (CVH) created a home monitoring program for enrolled patients with severe acute respiratory syndrome coronavirus 2 (SARS CoV-2) infection utilizing disease risk stratification and pulse oximeter readings to dictate nurse and clinician follow-up. We report raw data that compare surge levels in our community for the Alpha (B.1.1.7), Delta (B.1.617.2) and Omicron (B.1.1.529) surges.

**Methods:**

From April 2020 to present, the CVH enrolled patients diagnosed with COVID-19 based on FDA approved PCR tests. For this abstract, we defined any surge as an outpatient CVH census of greater than 50 patients or an inpatient census of >10 patients. We defined the maximum intensity of the surge as >20% outpatient SARS CoV-2 positivity and a CVH census >100 patients or >20 inpatients with SARS CoV-2 infection.

**Results:**

For the outpatient setting, days of surge and maximum intensity were 144 / 98 days for Alpha, 92 / 71 days for Delta, 74 / 47 days for Omicron, respectively. Average daily CVH admissions during surges were 17.7 for Alpha, 26.15 for Delta, and 27.15 for Omicron. Total emergency department (ED) and urgent care visits were 12,765 and 23,696 for Delta, and 9701 and 16102 for Omicron. In the inpatient setting, days of surge and maximum intensity days were 102/76 days for Delta and 78/48 days for Omicron. Our peak inpatient COVID-19 daily census was 51 and 50 patients for Delta and Omicron.

**Figure:**
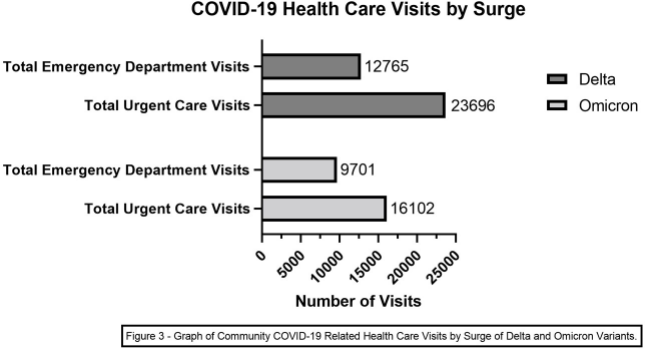

**Figure:**
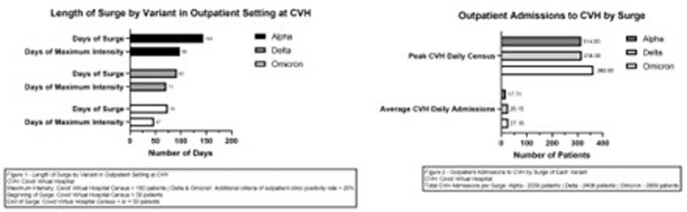

**Figure:**
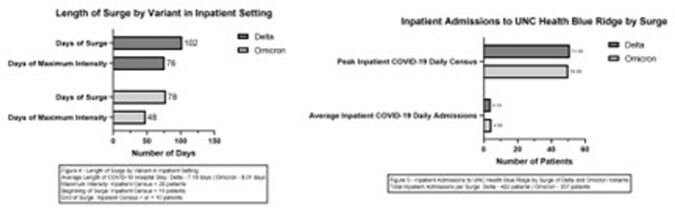

**Conclusion:**

Our CVH has enrolled over 8700 patients, and our hospital has not been on diversion during the COVID-19 pandemic, unlike many surrounding hospital systems of similar size. More importantly, the CVH has helped create a sustainable model that gathers local data to aid predictive algorithms and facilitate proactive rather than reactive resource allocations. This virtual model can be adapted for multiple other health conditions in the outpatient setting to improve patient safety and quality care.

**Disclosures:**

**All Authors**: No reported disclosures.

